# Intracoronary imaging-guided rotational atherectomy combined with intravascular lithotripsy in the treatment of severe coronary artery calcification—A case report

**DOI:** 10.3389/fcvm.2023.1184237

**Published:** 2023-06-09

**Authors:** Fengwen Cui, Yaliang Tong, Guohui Liu, Wenqi Zhang, Kun Liu, Daoyuan Si, Yuquan He

**Affiliations:** Department of Cardiology, China-Japan Union Hospital of Jilin University, Changchun, China

**Keywords:** severe coronary artery calcification, intravascular lithotripsy, rotational atherectomy, percutaneous coronary intervention, intracoronary imaging

## Abstract

**Background:**

Severe coronary artery calcification increases the difficulty of percutaneous coronary intervention procedures and impairs stent expansion. Herein, we report a case of a patient who was successfully treated with rotational atherectomy using a stepped burr strategy combined with intravascular lithotripsy for plaque modification under intracoronary imaging.

**Case summary:**

A 65 year-old woman presented to our hospital with recurrent chest pain evolving for 1 year. Coronary angiography showed approximately 80% stenosis of the proximal mid-left anterior descending artery. Intravascular ultrasound (IVUS) and optical coherence tomography (OCT) revealed a 360° annular calcification. The calcification was rotablated with 1.5 and 1.75 burrs, and the lesion was undilatable with a 3.0 mm non-compliant balloon at 14 atm. Subsequently, the intravascular lithotripsy was reset for the modification of the calcified lesion. A shockwave balloon measuring 3.0 mm × 12 mm was delivered, and 40 pulses were performed at 6 atm. Intravascular imaging modalities (IVUS and OCT) revealed a circumferential calcified plaque with deep fractures. After post-balloon expansion followed by drug-eluting stent placement with a final stent expansion of 84%, there were no intraoperative complications and no major adverse cardiovascular events within 90 days postoperatively.

**Conclusion:**

A combination of rotational atherectomy and intravascular lithotripsy may be an effective and complementary strategy for the treatment of severely calcified lesions that cannot be resolved using a single procedure. However, more clinical studies are required to clarify this finding.

## 1. Introduction

Severely calcified coronary lesions pose a specific challenge as balloon dilatation and stent placement can be difficult or even impossible, often leading to suboptimal stent expansion, which in turn is a major cause of stent restenosis and thrombosis. In 2020, the Society for Cardiovascular Angiography and Interventions published the procedure for calcification diagnosis and treatment, which suggested the use of rotational atherectomy (RA) and intravascular lithotripsy (IVL) in patients with severe calcification. However, at that time, IVL was not approved by the Food and Drug Agency (1). RA cannot disrupt the deep part of the calcified lesion, even if the size of the burr is upgraded. Nevertheless, IVL can be used to administer shock to the deep part of the calcified lesion and break it. The combination of these two techniques enables a more optimal endpoint for plaque modification. Herein, we report a case of a patient who was successfully treated with RA using a stepped burr strategy combined with IVL for plaque modification. We explored whether upgrading the rotational burr or applying IVL is the optimal treatment when the initial burr of RA cannot significantly modify the plaque.

## 2. Case description

**Figure 1:**
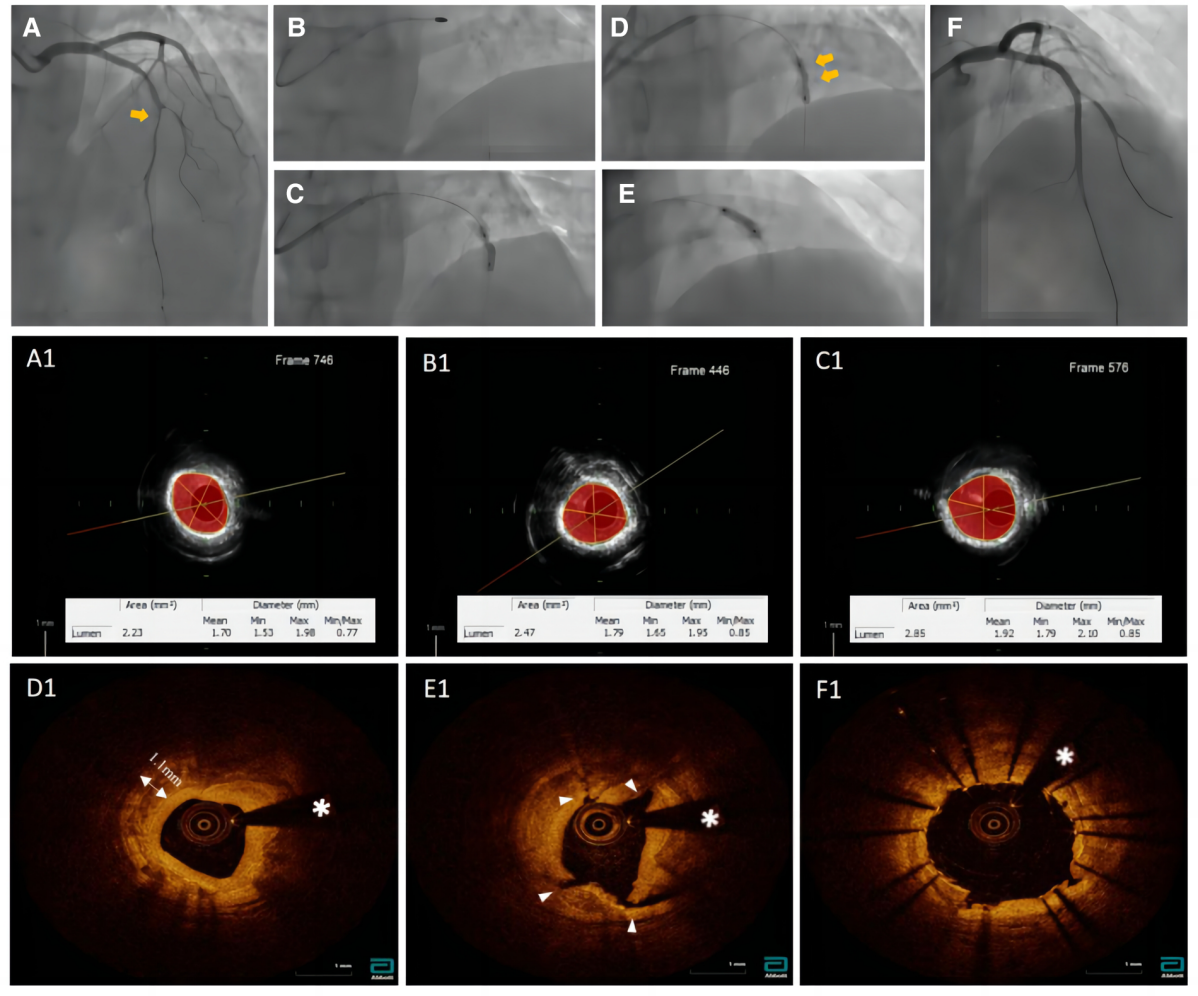
Pictures (**A1, B1, C1**) were taken with echoPlaque 3.0 (INDEC medical systems, Inc, mountain view, CA). (**A**) Baseline coronary angiogram. (**B**) Rotational atherectomy. (**C**) Failed dilation after 1.5 mm burr. (**D**) Angiogram after 1.75 mm burr and balloon dilation. (**E**) shockwave balloon. (**F**) Post-DES of LAD. (**A1**) The IVUS showed a 360° loop calcification. (**B1**) The IVUS imaging after 1.5 mm burr rotation. (**C1**) The IVUS imaging after 1.75 mm burr rotation. (**D1**) The OCT imaging showed no calcium fracture after rotational atherectomy procedure. (E1) Over three places of calcium fracture after IVL. (**F1**) Final OCT post stent.

**Figure 2:**
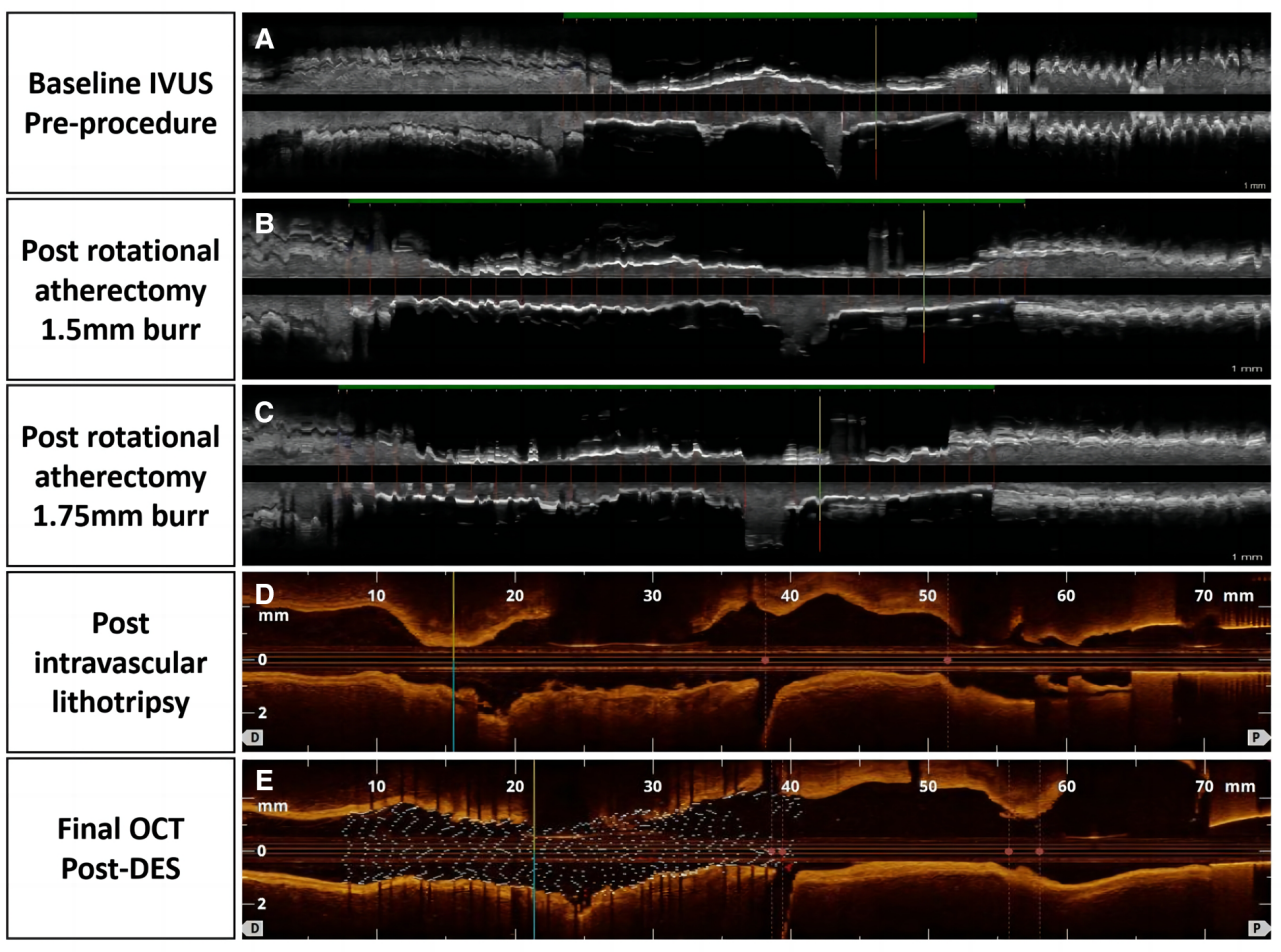
Tissue and lumen display mode, pictures (**A–C**) were taken with echoPlaque 3.0, pictures (**D**) and (**E**) showed longitudinal and 3D OCT views.

A 65 year-old woman with no hypertension, diabetes, or smoking history was admitted to China-Japan Union Hospital of Jilin University for recurrent chest pain evolving for 1 year. On admission, electrocardiography showed ST hypoplasia and T wave inversion in leads V1-V6. Cardiac ultrasonography revealed an ejection fraction of 64% with no structural changes. Blood analysis showed the following findings: cTn I, 0.02 ng/ml; NT-proBNP, 104 pg/ml; CHOL, 2.94 mmol/L; HDL-C, 0.96 mmol/L; and LDL-C, 1.55 mmol/L. Coronary angiography showed approximately 80% stenosis in the proximal mid-segment of the left anterior descending (LAD) artery and approximately 60% stenosis in the opening of the left diagonal artery (Figure 1A). Intravascular ultrasound (Boston Scientific Corporation, Maple Grove, MN) revealed calcification of the anterior descending artery in a 360° loop with a minimum lumen area (MLA) of 2.23 mm^2^ (Figure 1A1). A 1.5 mm burr of 150,000 r/s was applied to rotablate the LAD artery calcification (Figure 1B), and dilatation (2.75*15 mm NCB, Boston Scientific) was performed at 14 atm (Figure 1B1). The lesion clearly showed a “dog-bone” image (Figure 1C). After a stepped burr strategy with a 1.75 mm burr of 150,000 r/s, 3.0 mm × 12 mm non-compliant balloon (Boston) dilatation was performed (14 atm), and the lumen was enlarged without calcification fracture (Figure 1C1). At this point, the MLA was 2.09 mm^2^ (Figures 1D, D1). The maximum thickness and length of the calcification were approximately 1.1 mm and 21 mm, and calcium burden is 101%. A 3.0 mm shockwave balloon (Shockwave Medical, lnc, Betsy Ross Drive Santa Clara, CA) was applied to the LAD artery lesion (Figure 1E) at 6 atm with 40 pulses from far to near. Optical coherence tomography (Abbott Vascular, Santa Clara, CA, USA) showed a maximum fracture depth of 0.98 mm and an MLA of 2.77 mm^2^ (Figure 1E1). Finally, a drug-eluting stent (Boston Scientific) measuring 2.75 mm × 32 mm was implanted smoothly with a minimum stent area (MSA) of 5.51 mm^2^ and stent expansion rate of 84% (Figure 1F1). No significant calcification was observed at the diagonal artery, and non-compliant balloon (Boston) dilatation without stent placement was performed. Longitudinal reconstruction images showed the entire percutaneous coronary intervention procedure (Figure 2). The patient achieved hemodynamic recanalization with no intraoperative adverse events and no major adverse cardiovascular event (MACE) at the 90 day postoperative telephone follow-up.

## 3. Discussion

Coronary artery calcification increases with age, and approximately 73% of coronary lesions show calcification on IVUS (2). Severely calcified coronary lesions increase the difficulty and risk of interventions and remain an important cause of in-sent restenosis (ISR) and thrombosis after drug-eluting stent placement. No single procedure can solve all calcification-related problems. In our patient, intracoronary angiography showed a stenosis rate of 80%. However, angiography underestimates calcified lesions to a certain extent, and it is necessary to assess the severity and characteristics of the plaque using intracoronary imaging modalities. The patient had a 360° loop calcification with a maximum thickness and length of 1.1 mm and 21 mm, respectively, resulting in a calcium score of 4 (3). We performed RA using a stepped burr strategy and applied two burr sizes (1.5 mm–1.75 mm) successively for plaque treatment. A non-compliant balloon (NCB) was applied after expansion and rotablation, with no fracture of the calcification and no intraoperative adverse events such as entrapment or perforation. After the popularity of drug-eluting stents, RA has become significantly beneficial for the management of calcified plaques (4). RA is an effective treatment for most calcifications. With advances in the RA procedure, the incidences of slow flow and perforation have significantly reduced, with a recent study showing an incidence rate of 1.1%–1% (5). However, in cases of dramatic calcification, simple RA using a stepped burr strategy has limitations. Unlike RA that enhances superficial calcification modification, IVL characterized by circumferential modification has the potential advantage of uniform energy distribution (6), causing calcification breakage, in which the broken plaque remains in place and contributes to increasing vascular compliance as well as stent apposition and expansion (7). After rotablation, the calcification underwent a full and multiple fracture with IVL for plaque modification while using only one shockwave balloon guidewire and 40 pulses. Another advantage of IVL is its low complication rate. In the DISRUPT III study, the rate of serious intraprocedural complications was only 0.5% (8), whereas in the DISRUPT IV study, the rate of serious contrast-induced complications in the Asian population was 0% (9). According to a review (10), the incidence of MACE at 30 days was the same between the ROTAXUS and DISRUPT I trials; however, in terms of long-term outcomes, the 6- and 9 month incidences of MACE in the CAD I and ROTAXUS trials were 8.3% and 24.2%, respectively. IVL may have a lower incidence of long-term MACE than RA, and patients with severely calcified lesions often have more complex conditions such as hypertension, diabetes mellitus, chronic kidney disease, and cardiac insufficiency. Compared with a single procedure, a combination of RA and IVL seems to be more effective. After the proposition of the combined Rota-Tripsy (IVL + RA) procedure by Alfonso et al. (11), a case series of Rota-Tripsy for severe calcification was reported (12), demonstrating a good clinical effect of the combined procedure. The different modes of action of IVL and RA provide for a synergistic approach in certain lesions, with some requiring upfront RA by intimal calcium ablation to debulk the luminal calcium, thereby facilitating IVL balloon delivery; RA enables initial lesion modification and facilitates the passage of balloon catheters. IVL triggers intimal and medial calcium breakage, allowing for the final successful dilatation of heavily calcified lesions.

In most cases, initial NCB predilatation is sufficient to facilitate shockwave IVL access. However, if there is difficulty crossing the lesion with contemporary low-profile balloon catheters, RA will remain the first-line therapy (13). Furthermore, rotary abrasion is currently used more for plaque modification than plaque ablation (14). Minor burrs have also been recommended as an initial strategy for plaque modification (15, 16). RA can initially modify the intimal plaque, creating a pilot channel to advance the lithotripsy balloon. This achieves a successful deep calcium breakage, increasing luminal distensibility for proper stent expansion, which requires an objective assessment by IVUS/OCT. In most cases, if the IVUS/OCT guidewire can pass through the calcified plaque, then the shockwave balloon guidewire can also pass through the lesion without the need for pretreatment with RA. Moreover, Rota-Tripsy could be used as a bailout strategy to compensate for the disadvantage of each technique alone: in case of chronic total occlusion or enable the advancement of IVL balloon in long calcified lesions, allowing for the most reasonable use of the benefits of IVL in areas of circumferential calcification and deeper calcification thickness. In addition, Rota-Tripsy can be applied in ISR lesions. Kassab (17) reported the feasibility and safety of IVL for the treatment of ISR lesions in a series of 12 patients. Recently, with favorable procedural outcomes and a relatively high MACE rate within 1 year, Yasumura (18) also reported the procedural and clinical outcomes in 26 patients who underwent RA for undilatable ISR. Whether Rota-Tripsy will achieve different long-term results requires further exploration and validation.

**Figure 3:**
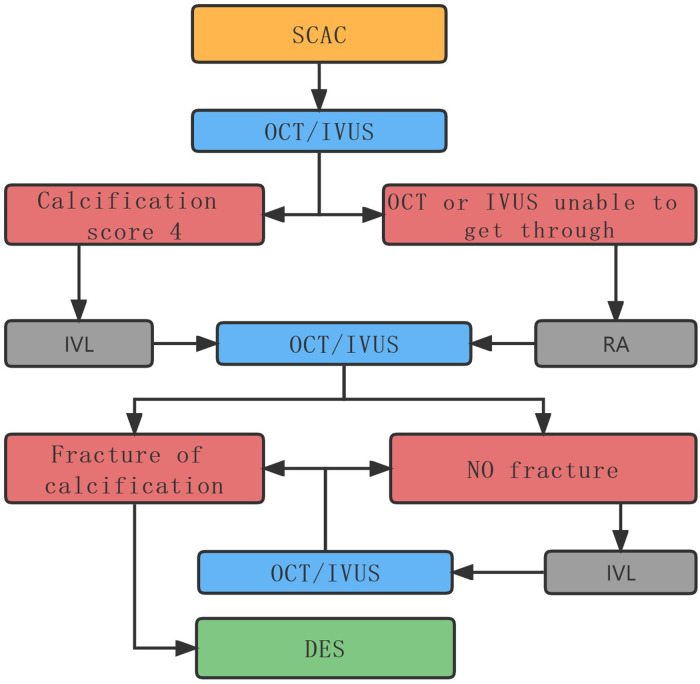
Management chart flow. SCAC: Severe coronary artery calcification.

Our patient achieved a good interventional outcome with no MACE occurrence at the 90 day postoperative telephone follow-up. Our case study provides insights and bases for improving the current management of severely calcified lesions (grade 4) (Figure 3). The application of IVL in combination with RA can help manage more complex coronary lesions.

In conclusion, although our aim was not to evaluate the outcome of severely calcified lesions in RA or IVL, in cases of severely calcified lesions that cannot be resolved with RA using a stepped burr strategy, the combination of RA and IVL is helpful.

## 4. Patient perspective

“I experienced no pain in my chest and improvement in my quality of life. Now I can do some houseworks without anxiety. I will insist on taking medicine.”

## Data Availability

The original contributions presented in the study are included in the article/Supplementary Material, further inquiries can be directed to the corresponding author/s.
