# Supplementary material for: Intracoronary imaging-guided rotational atherectomy combined with intravascular lithotripsy in the treatment of severe coronary artery calcification—A case report
Source: Front Cardiovasc Med. 2023 Jun 9;10:1184237. doi: 10.3389/fcvm.2023.1184237 (PMC10288993; doi:10.3389/fcvm.2023.1184237)

ID:
姓名:
性别: 女 年龄: 65 岁
科室/床号: /
门诊/住院号: /
检查编号:

HR: 69bpm PR: 187ms
QRS : 77ms
QT/QTcB/QTcF: 434ms/465ms/454ms
P/QRS/T : 5/44/-90deg
RV5+SV1 : 1.62+0.69=2.30mV
RV1+SV5 : 0.19+0.33=0.52mV

诊断提示:

诊断医生:

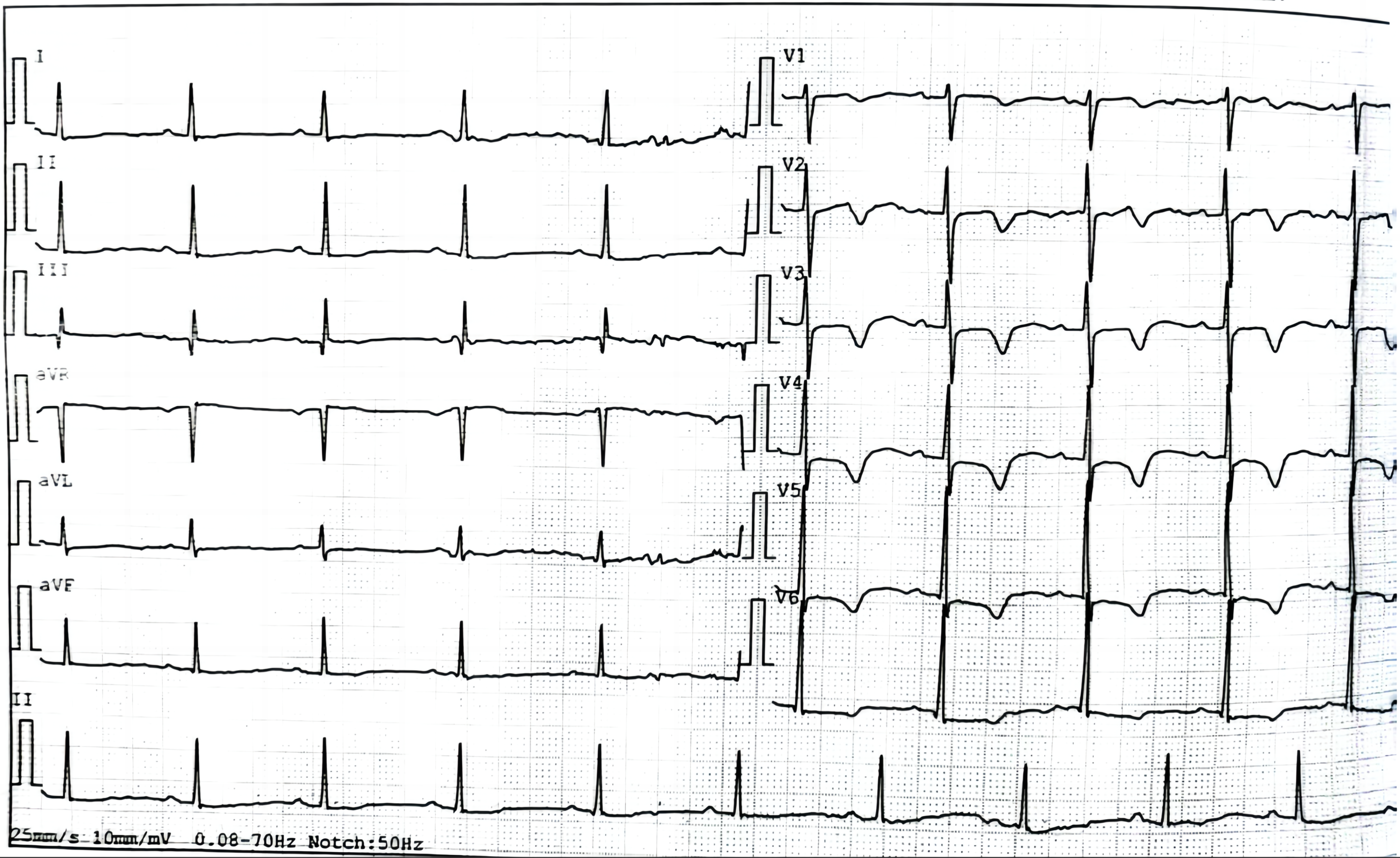

Supplement: Supplementary file 3 [file Datasheet1.pdf]
